# Retinal determination gene networks: from biological functions to therapeutic strategies

**DOI:** 10.1186/s40364-023-00459-8

**Published:** 2023-02-08

**Authors:** Shuangli Zhu, Wanling Li, Hao Zhang, Yuheng Yan, Qi Mei, Kongming Wu

**Affiliations:** 1 Department of Oncology, Tongji Hospital of Tongji Medical College, Huazhong University of Science and Technology, Wuhan, 430030 China; 2 Department of Geriatrics, Tongji Hospital of Tongji Medical College, Huazhong University of Science and Technology, Wuhan, 430030 China; 3 Cancer Center, Shanxi Bethune Hospital, Shanxi Academy of Medical Science, Tongji Shanxi Hospital, Third Hospital of Shanxi Medical University, Taiyuan, 030032 China; 4 Cancer Center, Tongji hospital of Tongji Medical College, Huazhong University of Science and Technology, Wuhan, 430030 China

**Keywords:** The retinal determinant gene network, DACH1, SIX1, EYA1, Cancer, Chronic kidney disease, Coronary artery disease, Organ development

## Abstract

The retinal determinant gene network (RDGN), originally discovered as a critical determinator in *Drosophila* eye specification, has become an important regulatory network in tumorigenesis and progression, as well as organogenesis. This network is not only associated with malignant biological behaviors of tumors, such as proliferation, and invasion, but also regulates the development of multiple mammalian organs. Three members of this conservative network have been extensively investigated, including DACH, SIX, and EYA. Dysregulated RDGN signaling is associated with the initiation and progression of tumors. In recent years, it has been found that the members of this network can be used as prognostic markers for cancer patients. Moreover, they are considered to be potential therapeutic targets for cancer. Here, we summarize the research progress of RDGN members from biological functions to signaling transduction, especially emphasizing their effects on tumors. Additionally, we discuss the roles of RDGN members in the development of organs and tissue as well as their correlations with the pathogenesis of chronic kidney disease and coronary heart disease. By summarizing the roles of RDGN members in human diseases, we hope to promote future investigations into RDGN and provide potential therapeutic strategies for patients.

## Introduction

The normal developmental programs, including cell proliferation, differentiation, morphogenesis, and tissue homeostasis, are regulated by many intra-cellular molecular regulatory networks [1, 2]. Generally, the coordination of these programs maintains the balance of physiological functions in the organism. Dysfunction of genes and signaling pathways in the developmental program will break the homeostatic balance and lead to the occurrence of disease, including tumors [3]. The retinal determinant gene network (RDGN) was initially discovered in the study of Drosophila eye specification and primarily consisted of dachshund (dac/DACH), so/SIX, eya/EYA, and the Pax6 homologs. Dac/DACH is a structural relative of the Ski/Sno gene family, a dominant suppressor of elipse; eya/EYA, a tyrosine phosphatase eyes absent; so/SIX, the SIX family transcription factor (TF) sine oculis; and the Pax6 homologs, eyeless (ey) and twin of eyeless (toy) [4, 5]. Many previous studies have found that this regulatory network is essential for the development of the mammalian organ system and tumor progression by encoding nuclear TFs and cofactors [6].

The DACH family is composed of DACH1 and DACH2 [7]. DACH1, a putative tumor suppressor, is involved in the oncogenesis of various cancers, such as breast cancer (BC), prostate cancer, and so on [8, 9]. Furthermore, DACH1 has also been implicated in a variety of developmental diseases, mainly including renal diseases and cardiac coronary arteries development diseases, such as chronic kidney disease (CKD), familial young-onset diabetes, pre-diabetes, coronary heart disease (CHD), and coronary arteriosclerosis [10, 11]. SIX, is a supergene family which includes SIX1-6 [12, 13]. SIX1 was found to synergize with the transcriptional cofactor EYA, which had tyrosine/threonine-phosphatase activity, to promote tumor initiation and progression [14, 15].

**Fig. 1:**
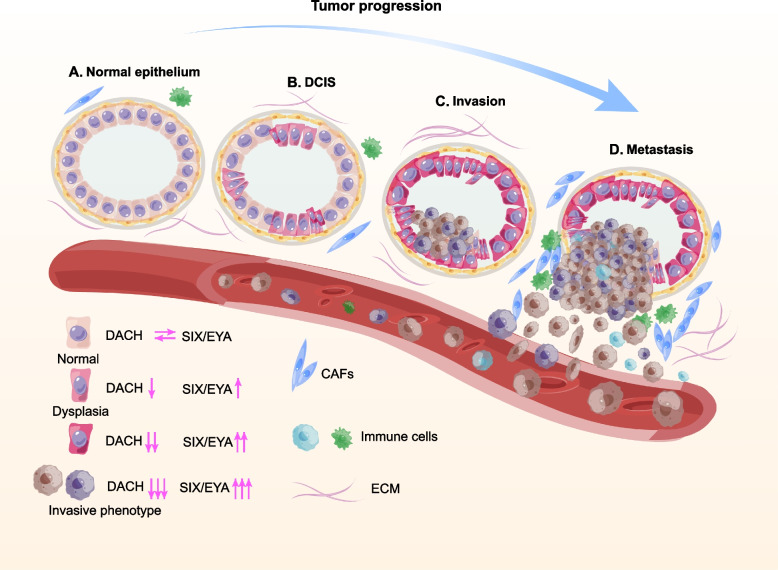
The Role of RDGN in the progression of breast cancer. The functional balance of DACH1 and SIX1/EYA keeps the homeostasis of luminal cell proliferation and apoptosis, which maintains the stability of the luminal structure. The abnormal expression of DACH1 and SIX/EYA caused a disorder of physiological functions. Subsequently, the luminal cells may undergo hyperproliferation, and transform to form ductal cancer in situ (DCIS). Then some cells acquire epithelial-mesenchymal transition (EMT) and trigger tumor metastasis by invading the basement membrane and entering blood vessels. “↓” means downregulated function, while “↑” means up-regulated functions. (The role of RDGN in the progression of RDGN was adapted from Fig. 1 in [16].)

It has been well demonstrated that the RDGN has been involved in tumorigenesis and the progression of BC. The balance of DACH1 and SIX1/EYA function keeps the homeostasis of luminal cell proliferation and apoptosis, which maintains the stability of the luminal structure. The abnormal expression of DACH1 and SIX/EYA caused a disorder in the balance of physiological functions [16]. Subsequently, the luminal may undergo proliferation, epithelial-mesenchymal transition (EMT), and progression to ductal carcinoma. It is generally accepted that a few malignant tumor cells, acquiring the features of cancer stem cells (CSCs) via EMT, trigger tumor metastasis by invading the basement membrane and entering blood vessels [16] (Fig. 1).

Here, we summarize the research progress on RDGN members from biological behaviors to therapeutic applications, especially emphasizing their functions in tumors. In addition, we discuss the roles of RDGN members in the development of organs and tissue, which are correlated with the pathogenesis of chronic kidney disease and coronary heart disease. By summarizing the functions of RDGN members in human diseases, we hope to promote future investigations into RDGN and provide new insights into therapeutic strategies for those diseases.

### The structures of RDGN members

RDGN has been discovered in the process of Drosophila eye determination. It attracted scholars' attention because it is a crucial signaling pathway in tissue specification and organogenesis of multiple organs. Furthermore, the abnormal expression and regulation of this network is linked to a range of different diseases, including cancers and congenital abnormalities or developmental defects [4, 5] (Table 1). This network mainly includes DACH, EYA, and SIX families.

**Table 1:** Summarization of targets regulated by RDGN

**RDGN member**	**Disease**	**Mechanism**	**Function**	**Reference**
**DACH1**	CRC (Organoid)	Interact with SMAD4 and inhibit BMP signaling	Inhibit cell proliferation, stemness and tumorigenesis	[17]
		Inhibit the expression of Wnt signaling downstream targets (c-Myc and cyclinD1)	Suppress CRC growth	[18]
		Inhibit TGF-β signaling	Inhibit migration and invasion of CRC	[19]
	BC	Down-regulating the transcription of MMP-9	Inhibit BC cell invasion and metastasis	[20]
		Inhibit the transcriptional activity of SNAI1	Repress breast carcinoma metastasis	[21]
		Decrease the level of CD44	Suppress BC progression	[22]
		Block YB-1	Represses YB-1-mediated oncogenic transcription and translation	[23]
		Bind to p53	Inhibit BC contact-independent growth	[24]
		Suppress IL-8	Inhibit BC cellular migration and invasion	[8]
		Inhibit TGF-β signaling	Inhibit TGF-β-induced apoptosis	[25]
	NSCLC	CXCL1	Predict poor prognosis of ADC	[26]
	ADC	CXCL5	Inhibit ADC invasion and tumor growth	[27]
		CXCL8	Repress tumorigenesis; improve prognosis	[28]
		Bind p53	Block the growth of ADC cells	[29]
		Downregulate of peroxiredoxin 3	Inhibit the proliferation and invasion of ADC	[30]
	EC	Activat TGF-β signaling	Inhibit esophageal cancer growth	[31]
		CXCL1/2	Inhibit NF-κB and reducing MDSC migration	[32]
	GC	Inhibit TGF-β signaling	Inhibit the invasion and metastasis of GC	[33]
	RCC	Inhibit cyclin D1 expression	Inhibit cellular proliferation and tumor growth	[34]
	HCC	Up-regulat p53 expression	Suppress the progression of HCC	[35]
		Reactivate TGF-β signaling	Suppress cellular growth	[36]
	endometrial carcinoma	Inhibite Notch1 pathway via c-Jun	Reverse MPA resistance and EMT	[37]
**DACH1**	OC	Inhibit TGF-β signaling	Inhibit OC progression	[38]
	Gliomas	Inhibit TGF-β signaling; IL-6; FGF	Inhibit tumor-initiating activity of glioma cells	[39]
	PC	CXCL1/2; CXCL5; CXCL8; IL-6; FGF	Inhibit PC cells growth and migration	[9, 40]
	Artery genesis	CXCL12	Stimulate shear stress-guided endothelial cell migration and coronary artery growth	[41]
		Inhibit the expression of FABP4, by endothelial Notch signaling	Result in the occurrence of cardiac hypertrophy	[42–44]
	diabetes	rs1408888 lies within regulatory elements of DACH1 implicated in islet development and insulin secretion	Cause familial young-onset diabetes, pre-diabetes	[11]
	Renal hypodysplasia	carrying homozygous missense mutations in both BMP4 (p.N150K) and DACH1 (p.R684C)	Cause renal hypodysplasia	[45]
**DACH2**	Myogenesis	Indirectly upregulated and activates Murf1 and Atrogin1 expression	Cause muscle atrophy	[46–48]
**SIX1**	BC	Activate TGF-β signaling	Induce properties of EMT	[49, 50]
		Activate MAPK and TGF signaling pathways	Mediate the accumulation of CSCs	[49]
	BC	Upregulate VEGF-C	Induce lymphangiogenesis and metastasis	[51]
	CC	Activate TGF-β signaling	Promote EMT, CSCs properties and malignant conversion	[52]
	CC	Upregulate VEGF-C	Promote tumor lymphangiogenesis	[53]
	EC	Activate TGF-β signaling	Promote tumor cells growth	[54]
	OC	Downregulate TRAIL	Cause resistance to TRAIL-mediated apoptosis and is associated with poor survival	[55]
	HCC	Bind to p53	Promote HCC progression	[35]
	HCC	IL-6/STAT3/MMP-9	Facilitate HCC cells invasion	[56]
	rhabdomyosarcoma	Activate cyclin D1 transcription	Lead to tumor initiation	[57]
	lung fibrosis	Increase the level of MIF	Promote lung fibrosis	[58]
	asthma	MMP-9; MMP-2	Suppress airway inflammation and remodeling	[59]
**SIX1**	erythroleukemia	Strengthen GATA1 function	Accelerate erythropoiesis	[60]
	AML	Block Wnt/SIX1 signaling	Suppress the progression of AML	[61]
	Myogenesis	SIX genes are expressed throughout muscle development	participate in the genesis and the maintenance of myofibers diversity	[62]
**EYA**	BC	Induce TGF-β signaling	Promote the metastasis of tumor cells	[63]
		Dephosphorylate PP2A-B55α	Increase cancer cells' metastasis capability	[64]
	cardiac hypertrophy	EYA2 activates mTOR signaling pathway	Cause cardiac physiological hypertrophy	[65]
		EYA4 mutation	Cause dilated cardiomyopathy	[66]
	BOR syndrome	mutation in SIX and EYA or disruption of the SIX/EYA complex	cause BOR syndrome	[67–69]

Abbreviations: *BMP* Bone morphogenetic protein, *CRC* Colorectal cancer, *CXCL* C-X-C motif ligand, *BC* Breast cancer, *MMP-9* Matrix metalloproteinase 9, *NSCLC* Non-small cell lung cancer, *ADC* Lung adenocarcinoma, *EC* Esophageal cancer, *GC* Gastric cancer, *RCC* Renal cell carcinoma, *HCC* Hepatocellular carcinoma, *TGF-β* Transforming growth factor beta, *MPA* Medroxyprogesterone acetate, *EMT* Epithelial to mesenchymal transition, *IL-8* Interleukin-8, *OC * Ovarian cancer, *PC* Prostate cancer, *MDSC* Myeloid-derived suppressive cell, *FGF* Fibroblast growth factor, *CC* Cervical cancer, *CSC* Cancer stem cells, *TRAIL* Tumor necrosis factor-related apoptosis-inducing ligand,  *AML* Acute myeloid leukemia, *PP2A* Protein phosphatase 2A, *BOR* Branchio-oto-rena

### DACH

**Fig. 2:**
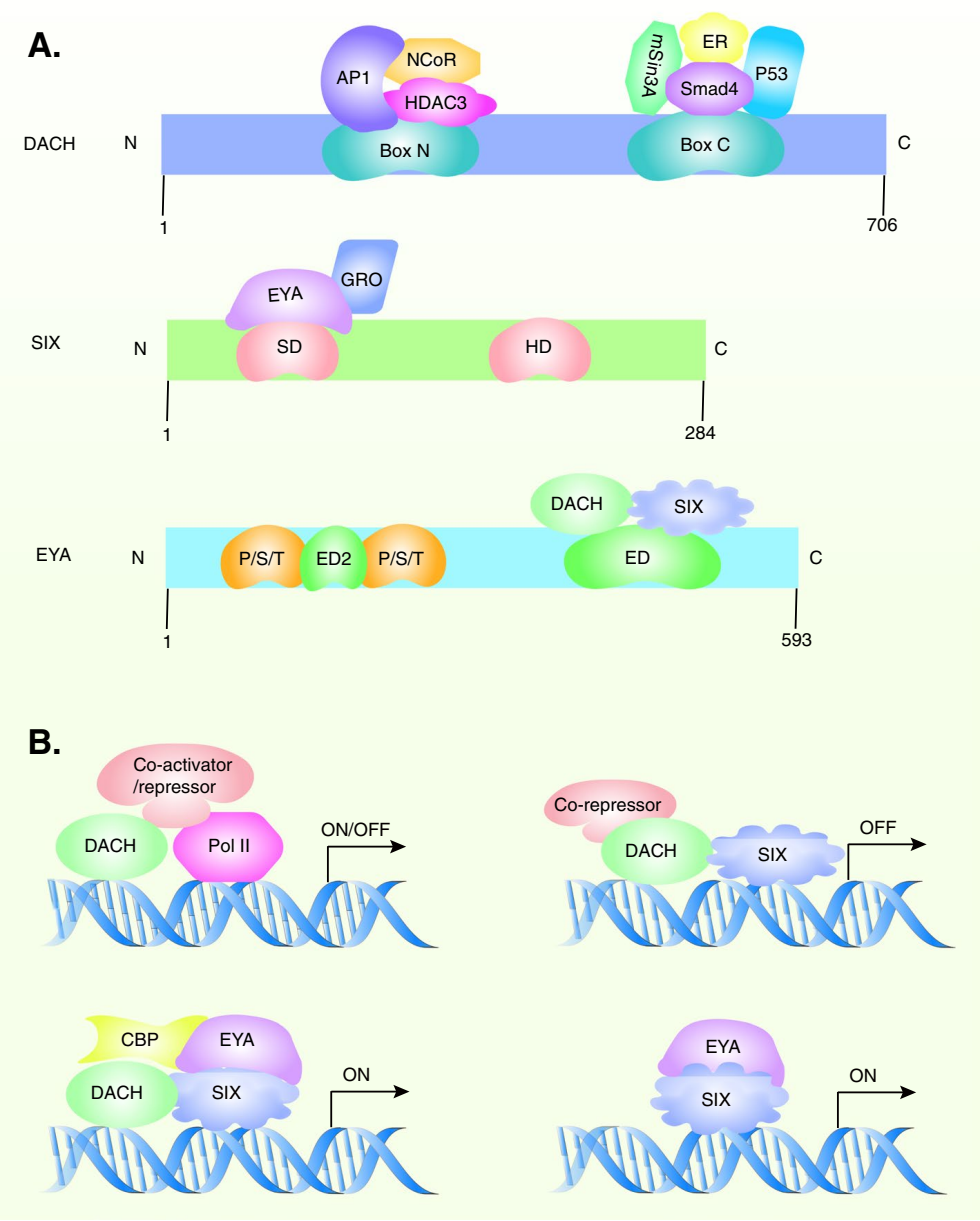
Structure of DACH, SIX, and EYA. **A** Key domain of DACH, SIX, and EYA. **B **The working model of DACH, SIX, and EYA cooperate with transcriptional activator/repressor and cofactor. Abbreviations: DACH, dachshund; EYA, eyes absent; ED, Eya domain; SD, Six domain; HD, Homeobox domain; ER, estrogen receptor; NcoR, nuclear corepressor; Gro, Groucho; HDAC, histone deacetylase; P/S/T, proline, serine and threonine rich region. (The structure of RDGN was adapted from Fig. 1 in [76].)

DACH is divided into two paralogs, including DACH1 and DACH2, encoding nuclear proteins with two deeply conserved domains: dachshund domain 1 (DD1) and dachshund domain 2 (DD2). DD1, also known as box-N, is able to bind to DNA. Another conserved domain is DD2, referred to as box-C, which is responsible for protein–protein interaction. Moreover, the X-ray crystal structure analysis has demonstrated that DD1 concludes a conserved domain, which is homologized with the Sno/Ski oncogene [7] (Fig. 2A). The DACH1 is capable of combining DNA sequences and chromatin or binding to other TFs (such as c-Jun, Smads, SIX, and estrogen receptor-α (ER-α)), which lead to the inhibition of the specific target genes transcription [14, 70–72]. Besides, the DACH1 DNA-binding site, which resembled the binding site of FOX (forkhead box-containing protein), competitively bound to FOX and inhibited forkhead signaling. Therefore, DACH1 replaced the function of FOXM1 in the promoter of the target gene and competed with FOXC2 to inhibit the expression of genes involved in the invasion and metastasis of tumor cells [71, 73, 74]. Moreover, it's reported that DACH1 remodels chromatin by the recruitment of NcoR, Sin3, and HDACs or CBP, resulting in the suppression or activation of transcription [25, 73, 75].

DACH1 is not only involved in tumor progression but also participates in CKD, cardiovascular diseases, and bilateral cystic renal dysplasia [10, 11, 45]. Although the significance of DACH1 in human diseases has been widely investigated in the past decades, the roles of DACH1 in disease pathogenesis and the underlying mechanisms remain largely unknown.

### SIX

SIX gene family, a homeobox gene superfamily, plays a role in tumor initiation and progression and organ development by encoding TFs to activate or inhibit the transcription of downstream target genes [77]. SIX family members consist of the SIX1/2, SIX3/6, and SIX4/5 superfamilies, which correspond to the homologs of sine oculis, optix, and D-SIX4, respectively [12, 13]. Similar to DACH1, SIX family also has two highly conserved sequences, including the homeoprotein domain (HD) and the SIX domain (SD) [67, 78]. The HD is related to the DNA binding process, and SD is associated with the interaction of protein–protein [67] (Fig. 2A). Moreover, A series of studies have shown that SIX2, SIX4, and SIX5 had endogenous transcriptional activation domains in addition to SD and HD. Therefore, co-activator proteins (such as EYAs) were not necessary for the transcription of these proteins [79, 80]. However, SIX1, SIX3, and SIX6 required co-activator (such as EYA1-4) to stimulate their transcriptional activity [81]. It’s well accepted that SIX proteins could promote tumor growth and other tumor biological behaviors [82, 83]. Additionally, it was reported that EYA is a co-activator of SIX while DACH is a co-suppressor of SIX [14, 76] (Fig. 2B). Hence, RDGN members play a coordinate role in the process of tumorigenesis.

### EYA

The EYA family is composed of EYA1-4, most of which are transcriptional cofactors [84] (Fig. 2A). It has been indicated that the EYA family plays a role in the regulation of transcription and signal transduction processes owning to it containing three separate biochemical activities: tyrosine phosphatase, threonine phosphatase, and transactivation [85]. EYA has two tyrosine phosphatase activity targets, including histone variant H2AX [86, 87] and ERβ Y36 residue [88]. By dephosphorylating H2AX, EYAs such as EYA1, EYA2, and EYA3 induce DNA repair in cells after DNA damage [86, 87]. EYA1 stimulated the activity of Cyclin D1, and EYA2 inhibited the transcriptional activity of the ER-β by dephosphorylating the Y36 residue, thus promoting cancer cellular proliferation and tumor growth [88, 89]. EYA4 has threonine phosphatase activity and has a significant role in the innate immune response [90]. As a member of the RDGN family, EYA functions by regulating transcription and tyrosine phosphatase activity. The abnormal expression or activity of EYA is related to tumorigenesis, such as Ewing sarcoma and lung adenocarcinoma [91–93]. Moreover, mutations of SIX1 or EYA contribute to branchio-oto-renal (BOR) syndrome, muscle defects, asthma, etc. [59, 94, 95].

### The role of RDGN in cancer

It is well recognized that DACH1, SIX, and EYA, the key members of RDGN, have abnormal expression in various cancers [76, 96]. RDGN played a key role in tumor initiation and progression by interacting with multiple signaling pathways in a coordinated manner, regulating the proliferation, apoptosis, movement, and stemlike property [63, 76, 96–98]. In general, DACH1 is down-regulated as a tumor suppressor, while SIX and EYA are up-regulated in tumors, which promotes tumorigenesis and tumor progression [99]. Watanabe et al. proposed that DACH1 could be a unique tumor suppressor [39]. A comprehensive and deeper understanding of how RDGN works in tumors will contribute to new therapeutic approaches by targeting RDGN members.

### Proliferation

**Fig. 3:**
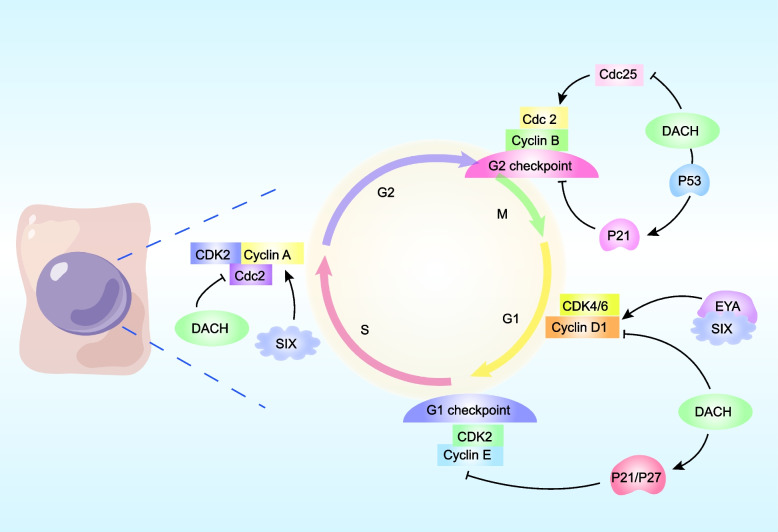
The Role of RDGN in the Cell Cycle. DACH1 inhibits cyclin D1 transcription, resulting in G1-phase arrest. SIX1/EYA facilitates G1 progression via promoting the transcription of cyclin D1. Moreover, DACH1 also inhibits cyclin A, but SIX1 enhances cyclin A in S-phase. DACH1 increases the protein abundance of p21 and p27, further suppressing cdk2/cyclin E activity and finally preventing cells from entering S-phase. DACH1 also binds with p53 and synergizes p21 induction, and inhibits Cdc25, reducing the activity of Cdc2 to arrest cells in G2/M. (The role of RDGN in the cell cycle was adapted from Fig. 2 in [76].)

The dysregulation of mitosis signals is one of the prominent features of cancer cells, which leads to a disordered cell cycle and abnormal proliferation and eventually results in tumor initiation and progression [100, 101]. The RDGN maintained the dynamic balance of cells in the progression of the cell cycle (Fig. 3). Overexpression of DACH1 promoted renal cell arrest in the G1/S or G2/M phase by downregulating cyclin D1 and cyclin A and upregulating P21 and P53 [102]. Further analysis proved that DACH1 inhibited the transcription of cyclin D1 and cyclin A by directly binding to its corresponding promoter region, thereby further repressing the proliferation of cells and tumor growth in various cancer types, such as BC and renal cancer [34, 70, 103–105]. However. Lee et al. found that DACH1 induced cyclin D1 expression while inhibiting P21 expression in myeloid progenitor cells [106], which is fairly contradicted by previous findings in solid tumors. We speculate that there may exist significant functional differences between solid and hematologic tumors, as well as between progenitor and differentiated cells. The clinical database showed that the expression of Kruppel-like factor 5 (KLF5) was positively correlated with Y-box binding protein 1 (YB-1) while negatively correlated with DACH1 in patients with BC. Furthermore, YB-1 regulated KLF5 expression by inhibiting DACH1 transcriptional activation or stabilizing KLF5 mRNA construction, resulting in the progression of triple-negative breast cancer (TNBC). Ribosomal S6 kinase 2 (RSK2) mediated YB-1 phosphorylation, promoted the formation of the YB-1/KLF5 transcription complex, and further enhanced the proliferation of tumor cells. Consequently, the RSK inhibitor LJH685 primarily inhibited BLBC tumorigenesis by interfering with the YB-1-KLF5 axis ​[107].

Xu et al. confirmed that SIX1 was highly expressed in rhabdomyosarcoma cells, inhibited rhabdomyosarcoma cell differentiation, and promoted tumor growth in vivo through MYOD1 and MYOG-mediated transcriptional genomic changes [108]. SIX1 promoted cyclin D1 transcription in rhabdomyosarcoma cells, leading to tumor initiation [57], and SIX1 also facilitated BC cell proliferation via inducing cyclin A expression [109]. Furthermore, EYA regulates the cell cycle and consequently promotes BC cell proliferation by increasing cyclin D1 transcription [89].

In addition to changing cyclin D1 expression, RDGN also regulates cell cycle progression via affecting P53 transcription. DACH1 binds to P53, enhancing P53-mediated cell cycle arrest to inhibit tumor growth [24, 29]. In contrast, SIX1 negatively regulates P53 to promote BC cell proliferation through upregulating microRNA-27a-3p and downregulating ribosomal protein L26 (RPL26) [110]. A recent study showed that SIX1 knockdown promoted DACH1 expression in vitro and in vivo in hepatocellular carcinoma (HCC) cells, which synergistically induced P53 expression and inhibited HCC progression [35]. The imbalance between the tumor-suppressor function of DACH1 and the oncogenic effect of SIX/EYA not only accelerates cell cycle disorder but also decreases apoptosis, providing the basis for tumorigenesis.

CSCs promote tumor progression and induce immune evasion through facilitating angiogenesis, proliferation, and colonization in other sites, which are strongly related to therapeutic resistance [111–113]. It has been demonstrated that RDGN is involved in the regulation of CSCs, especially in BC. Counteracting transcriptional activation of NANOG, KLF4, and SOX2, DACH1 reduced the proportion of breast CSCs [97]. The overexpression of SIX1 increases phenotypic CSCs in some cancers, such as breast, esophageal, colorectal, and pancreatic cancers [49, 54, 114, 115]. SIX1 mediate the CSCs phenotype by activating MAPK and TGF signaling pathway [49, 52]. Similarly, EYA also increases the proportion of breast CSCs via phosphatase activity [89]. Thus, RDGN may be a potential target to address therapeutic resistance by reducing the proportion of stem cells.

### Invasion and metastasis

**Fig. 4:**
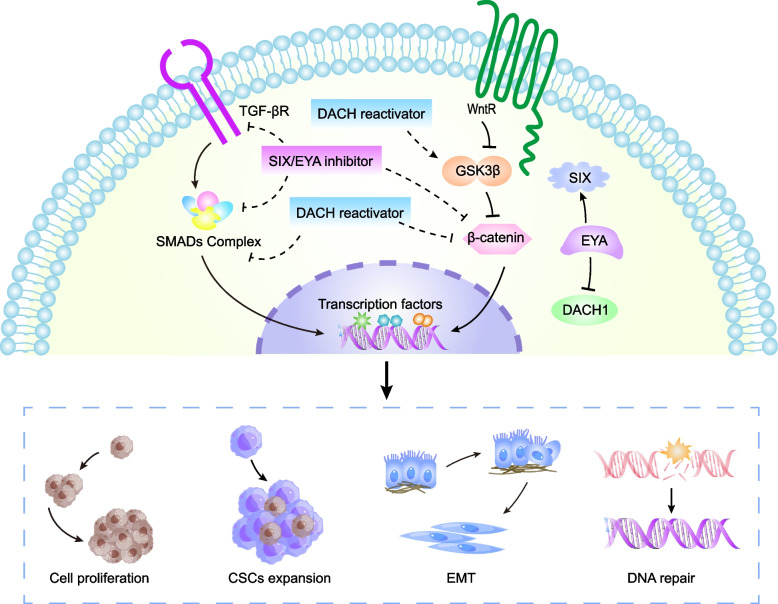
The interaction of RDGN with key signaling in cancer. TGFβ and Wnt signaling pathways are vital for tumorigenesis and progression. RDGN is involved in the regulation of TGF-β and Wnt signaling pathways. DACH1 inhibits smad complex, while SIX/EYA activates TGF-ßR. DACH1 represses ß -catenin, while SIX/EYA activates Wnt signaling. EYA could switch DACH1 repressional function and enhance activation of SIX. Silencing the expression of SIX or interfering with the SIX/EYA complex using small molecule compounds can block the function of SIX. Moreover, EYA phosphatase activity can be inhibited by biochemical inhibitors. The use of demethylating agents can reactivate DACH1 expression. Therefore, targeting RDGN members holds promise as a cancer treatment strategy

EMT is a dynamic process in which cells lose their epithelial phenotype and acquire the characteristics of a mesenchymal phenotype [116]. EMT renders cells high mobility, thus contributing to tumor cell migration and invasion [117]. Tumor cells detach themselves from the primary tumor site to colonize the secondary sites (distant organs), forming metastasis [118]. EMT is induced by activating Wnt/TGF-β signaling, which enhances the ability of tumor cells to migrate and invade [119–122]. Many studies demonstrated that RDGN is involved in the process of EMT through mediating Wnt/TGF-β signaling pathway [6] (Fig. 4).

DACH1 indirectly activates E-cadherin via inhibiting the transcription of SNAI1 in BC, which suppresses tumor cell migration and invasion [21]. Similarly, Bu et al. demonstrated that DACH1 inhibited pancreatic cancer cell invasion via activating E-cadherin and repressing N-cadherin and vimentin [123]. Furthermore, DACH1 suppressed tumor growth and EMT of mouse BC models via blocking YB-1 [23] and inhibited BC cell initiation by decreasing CD44 levels [22]. In addition, DACH1 suppressed MMP-9 transcription to reduce BC invasion and metastasis [20]. Overexpression of DACH1 inhibited the TGF-β-mediated EMT, thus suppressing colorectal cancer (CRC) cell growth, invasion, and metastasis. These results suggest that DACH1 is a prospective target for cancer therapies [19].

SIX1 also plays an important role in tumor invasion and metastasis. The upregulation of SIX1 was related to poor prognosis in many malignancies, such as cervical cancer, CRC, prostate cancer, and so on. Kong et al. found that SIX1 facilitated epithelial cancer cell proliferation, migration, and invasion via mediating the VEGF-C/NRP2/GLI signaling axis [124]. The knockdown of SIX1 suppressed the process of EMT in papillary thyroid carcinoma through inhibiting the TGF-β/SMAD2/3 signaling pathway [125]. Meanwhile, Huang et al. demonstrated that SIX1 facilitated migration and invasion in non-small cell lung cancer via activating the Notch pathway. The γ-secretase, a Notch pathway inhibitor, reversed the SIX1-mediated phenomenon [126]. Intriguingly, SIX1 was found to facilitate the EMT process in BC by forming a negative feedback loop with miR-204-5p [127]. Micalizzi et al. found that SIX1 contributed to BC cell invasion and metastasis via regulating TGF-β-induced EMT [128]. SIX1 promotes EMT in cervical cancer cells via the TGF-β/SMAD signaling pathway, in contrast to DACH1 [50, 129]. By enhancing NF‐κB/p65 pathway in macrophages, SIX1 enhanced the expression of matrix metalloproteinase 9 (MMP-9) and further promoted the invasion of cancer cells. Moreover, SIX1 triggered the activation of the IL-6/STAT3/MMP-9 signaling pathway, which further facilitated HCC invasion [56]. In CRC, it has been found that overexpression of SIX1 promotes cell invasion by mediating the expression of TEAD4 and ZEB1, or inhibiting miR-200 expression [130–132]. Furthermore, SIX1 contributed to the proliferation, invasion, and EMT of gastric cancer cells via multiple pathways, including cyclin D1, ERK, MMP-2, and E-cadherin [133]. Similarly, the up-regulation of SIX1 expression in cervical cancer induces EMT to enhance the proliferation, invasion, and migration of cells [134]. A study has shown that SIX1 promoted EMT and stem cell phenotype, leading to the activation of the Wnt pathway, such as cyclin D1 [128]. Smith et al. indicated that SIX1 activated the pro-tumor TGF-β signaling through regulating the expression of the miR-106b-25 cluster. At the same time, the miR-106p-25 cluster also could induce tumor cell EMT [135]. CircNHSL1 was shown to be highly expressed in gastric cancer tissues and cell lines and was associated with poor prognosis in patients. Mechanistically, circNHSL1 promoted gastric cancer invasion and metastasis by acting as a miR-1306-3p sponge to derepress its target SIX1. Meanwhile, SIX1 directly binds to vimentin promoter to further enhance vimentin expression This result suggested that CircNHSL1 promoted gastric cancer progression through the miR-1306-3p/SIX1/Vimentin axis [136].

As mentioned above, EYA function as a co-factor for SIX1. EYA enhanced the stemness of cancer stem cells and induced EMT by synergizing with SIX1 [137]. SIX1 in cooperation with EYA2 promoted tumor cell metastasis by inducing TGF-β signaling and EMT [63]. Zhang et al. found that EYA3 enhanced the stability of c-Myc via the dephosphorylation of protein phosphatase 2A-B55α, which increased the metastasis capability of BC cells [64]. Moreover, other studies also showed that EYA3 stabilized Myc through threonine phosphatase activity to dephosphorylate Myc at pT58, then MYC upregulated PD-L1 and decreased CD8^+^ T cell activity. Given the immunosuppressive role of EYA3, w inhibition of EYA3 may enhance the effect of immune checkpoint therapy [138].

However, there are some contrary reports on RDGN’s function in different cancers. DACH1 was up-regulated in ovarian cancer, which might facilitate the resistance to the TGF-β signaling pathway. Moreover, DACH1 protein levels were increased with the invasiveness of ovarian cancer and the subcellular distribution of DACH1 changed from nucleus in normal tissue to cytoplasm in cancer [38, 139]. Zheng et al. demonstrated that SIX3 was downregulated in numerous cancers, such as breast, glioma, lung adenocarcinoma, and gastric cancers [140–143]. As a negative regulator of the Wnt pathway, SIX3 inhibited breast cancer carcinogenesis and metastasis by recruiting the LSD1/NuRD (MTA3) complex. Moreover, expression profile analysis indicated that high SIX3 mRNA level was a protective factor for OS and RFS of basal-like breast cancer patients [141]. EYA4 functions as a tumor suppressor in pancreatic ductal adenocarcinoma (PDAC) by inhibiting the activation of β-catenin and ID2, representing a favorable prognostic factor in PDAC [144]. Moreover, EYA4 was also associated with a favorite prognosis in HCC and intrahepatic cholangiocarcinoma [145, 146].

Therefore, RDGN plays a different role in various cancers, and further exploration will clarify the organ/tissue-specific function of RDGN in cancer.

### The reciprocal regulation of RDGN components

Recent studies have shown that there is a crosstalk among the components of RDGN and RDGN work coordinated to regulate tumor progression. The work by Cheng et al. found that the overexpression of DACH1 inhibited tumor initiation via up-regulating p53 expression, while SIX1 enhanced tumorigenesis via downregulation of p53 expression. Consistently, it was observed that the knockdown of SIX1 enhanced the expression of DACH1, which further activated the expression of p53 and ultimately inhibited hepatocellular carcinoma progression [35]. In addition, Farabaugh et al. showed that in BC, SIX1 cooperated with its co-activator EYA2 to induce the activation of the TGF-β signaling pathway, thereby promoting the malignant biological behavior of cancer cells, such as EMT, and the proliferation of cancer stem cells [63]. Similarly, SIX1/EYA signaling also transformed normal hematopoietic progenitors into leukemia [147]. SIX1 up-regulated VEGF-C expression and promoted lymph angiogenesis and lymphatic metastasis. Moreover, SIX1 induced the transcription of VEGF-C by enhancing the activation of the TGF-β/SMAD signaling pathway [51, 148]. In papillary thyroid cancer tissues, protein expressions of SIX1 and EYA1 are positively correlated, SIX1 stabilizes EYA1 at the post-transcriptional level and activates STAT3 signaling requiring EYA1 [15].

Crystal structure study showed that SIX1 helix interacted with HAD catalytic domain of EYA, and SIX1-EYA interaction was required for the activation of TGF signaling and EMT-like phenotypes. Moreover, disruption of SIX1-EYA interaction abolished SIX1-induced metastasis [67]. SIX1 was highly expressed in meningiomas. SIX1 and EYA2 functioned together to support the expression of leptin receptors, which promoted the proliferation of meningioma patient-derived cell lines [149]. Exploring the interaction of RDGN will help us better understand its role in tumors and the facility to develop effective therapeutic strategies.

### RDGN in other diseases

**Fig. 5:**
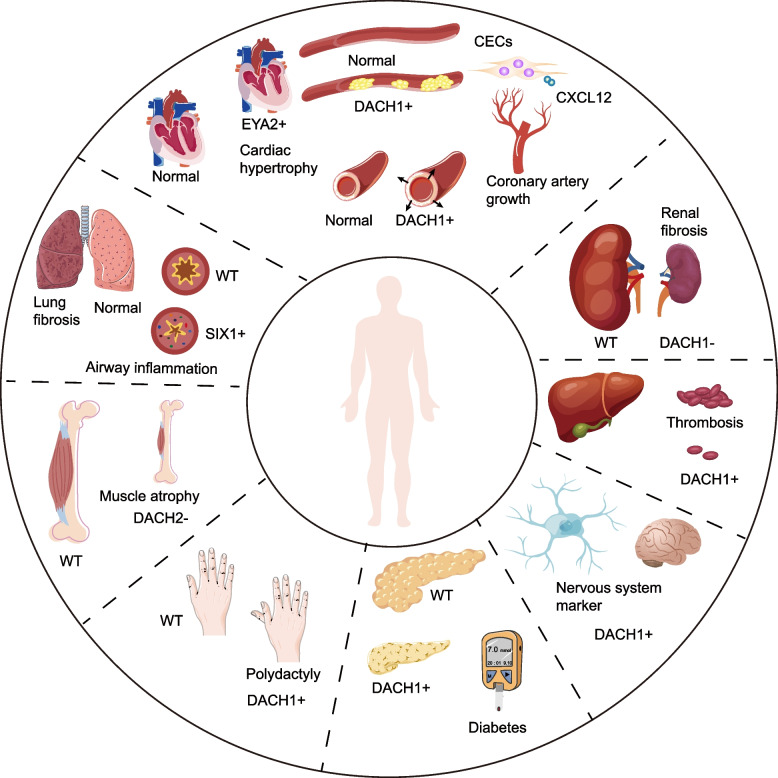
The role of RDGN in human diseases. DACH1 is required for the elongation of the coronary artery. DACH1 stimulates coronary artery endothelial cells (CECs) proliferation and promotes the migration of CECs by up-regulating CXCL12. Moreover, the overexpressed DACH1 in capillary endothelial cells promotes arterial normalization and prolongs arterial branches. The overexpression of EYA2 directly activates mTOR, leading to cardiac hypertrophy. In the hormonal system, DACH1 is linked to islet development, and insulin secretion, proving that DACH1 is involved in the pathogenesis of type-2 diabetes. In the urinary system, knocked down the expression of DACH1 caused damage to the kidney podocytes and tubular cells, eventually leading to CKD. Moreover, DACH1 knockout mice are more likely to aggravate the progress of diabetes nephropathy. DACH1 inhibits the expression of ATF6 and reduces tissue-type plasminogen activator (tPA) in mouse liver cells, reducing the risk of thrombosis. In the nervous system, DACH1 is a specific marker for neuroepithelial and ventricular radial glial cells. DACH1 is identified as one of the driver genes for postaxial polydactyly in the locomotor system. After denervation, lower DACH2 is observed in adult muscle fibers, allowing myogenin and nAchR expression to be up-regulated. While overexpression of DACH2 in denervated muscle reduced Mgn, nAChR, and MuSK gene induction depending on HDAC inhibitor. In the respiratory system, overexpression of SIX1 increases macrophage migration inhibitory factor (MIF) levels and promotes pulmonary fibrosis. Six1 is up-regulated in the chronic asthma mouse model. Silencing of Six1 reduced the expression and secretion of the airway remodeling-related mediators MMP-2 and MMP-9 through inhibition of the NF-KB pathway

In addition to the function of RDGN in tumors, recent studies have found that RDGN plays an important regulatory role in the occurrence and development of multiple human diseases (Fig. 5).

### Kidney

Diabetes and hypertension are the most common CKD risk factors [150, 151]. Moreover, it’s well-accepted that diabetic kidney disease (DKD) is one of the crucial causes of end-stage renal disease (ESRD) [151]. With the transcriptomic studies of human DKD and transcriptome-wide association studies of human CKD [38, 139], DACH1 in glomerular and tubular cells was demonstrated to be a protective factor for their function [152, 153].

The CKDGen consortium identified DACH1 as one of 13 novel loci influencing renal function and CKD through genome-wide association studies [10]. And DACH1 has also been identified as a gene with a therapeutic intervention function in CKD. In murine folate and diabetic kidney injury models, mice with tubule-specific DACH1 deletion were more susceptible to severe renal fibrosis. In contrast, fibrosis was rarely observed in mice with renal tubular-specific overexpression of DACH1. These results indicated that DACH1 could be used as a risk gene for kidney disease [152]. DACH1 regulated podocyte cell cycle and podocyte structure and function. Reduced DACH1 expression is associated with albuminuria and CKD [154]. DACH1-deficient mice were susceptible to hyperglycemia, streptozotocin, and adriamycin-induced kidney injury. Podocyte-specific overexpression of DACH1 protects mice from diabetic kidney disease. However, the mechanisms that reduce the expression of DACH1 are unknown [153]. Davis also found that mice with loss of function mutation in DACH1 exhibit postnatal lethality associated with respiratory dysfunction [155]. Meanwhile, one case report reported that the patient with concomitant mutations in DACH1 and BMP4 developed renal dysplasia [45]. Now it is well accepted that DACH1 protects podocyte and tubular cells from injury expression and its expression correlates with kidney function [156]. Considering that DACH1 is a protective factor for CKD, we speculate restoring DACH1 to normal levels may be a therapeutic target for kidney injury.

SIX1 and SIX2 are expressed in human fetal nephron progenitor cells. Although 80% of the target sites of SIX1 are shared with SIX2, and the expression of SIX1 in the posterior renal mass spectrometry system overlaps with SIX2, SIX1 couldn’t compensate for renal phenotype for the SIX2 deficiency. These results indicated that there were differences between SIX1 and SIX2 in the physiological role of kidney development stems from the transcriptional regulation of genes and the different biochemical characteristics of proteins [157].

### Cardiovascular system

RDGN also plays a vital role in the cardiovascular system. Chang et al. found that the mice lacking endothelial DACH1 could lead to the reduction of coronary artery diameter and overexpression of DACH1 stimulated endothelial cell polarization and migration predominantly by up-regulating CXCL12/CXCR4 signaling [41]. Further study indicated that overexpression of DACH1 in capillary endothelial cells promoted coronary artery differentiation and prolonged arterial branches. In addition, the overexpression of DACH1 enhanced the ejection fraction and cardiac function, and improved survival in myocardial infarction mice [42]. Ischemic heart disease is majorly caused by atherosclerotic plaque or thrombotic vessel occlusion to block the blood supply. The observation that DACH1 stimulates arterial regeneration and improves cardiac function support a potential therapeutic approach by modifying the DACH1 signaling in heart endothelial cells.

### Nervous system

DACH1 is involved in the development of the nervous system. The Castiglioni team found that DACH1 was overexpressed in proliferating neural progenitor cells in developing ventricles, subventricular regions and striatum from 5–21 weeks post-conception human brain samples. Single-cell transcriptome analysis identified DACH1 as a specific marker for neuroepithelial and ventricular radial glial cells [158, 159]. Genome-wide association study suggested that there was an age-dependent association between DACH1 and cerebral white matter volume [158, 159]. Antineuronal nuclear antibodies, a kind of novel neural autoantibody, are used as a biomarker of paraneoplastic neural autoimmunity [160]. Zekeridou et al. reported that DACH1 could be used as the autoantigen of ANNA3 determined by immunohistochemical colocalization and antigen specificity assay, and DACH1-IgG was identified as a new biomarker of neural autoimmunity [161].

### Hepatocyte

The expression of DACH1 was reported to be increased in the liver of obese mice and humans. Liver-specific silencing of DACH1 improves hepatocyte insulin signaling, and protects against hyperinsulinemia, which suggested that DACH1 was also involved in the metabolism of hepatocytes. Molecular analysis demonstrated that silencing of DACH1 in the hepatocytes increased the expression of ATF6 by inhibiting the nuclear rejection of calcium/calmodulin-dependent protein kinase II (CaMKII) or histone deacetylase 4 (HDAC4) [162]. Tissue-type plasminogen activator (tPA) prevents excessive coagulation without compromising hemostasis. ATF6 is an inducer of tPA and inverse correlation between DACH1 and ATF6 in the human liver. Increased circulating tPA, fibrinolytic activity, and bleeding time were observed in DACH1-knock-out mice, which can be reversed by silencing ATF6 [163]. The balance between plasma PAI-1 and tPA proteins controls the fibrinolysis. DACH1 expression in hepatocytes between obese and lean affects the balance though regulating ATF6 ​[164]. Those studies provide a prospective therapeutic method to reduce the risk of thrombosis and improve fibrinolysis.

### Development of limb, ear, and muscles

Polydactyly is a common congenital limb anomaly disorder caused by patterning defects in the developing anterior–posterior axis [165].​ DACH1 was identified as one of the causative genes for postaxial polydactyly by whole-exome sequencing and Sanger sequencing of DNA from individuals with postaxial polydactyly [166].​

Branchio-oto-renal syndrome (BOR) is characterized by hearing loss, and craniofacial and/or renal defects. So-binding protein (Sobp) binds as a SIX1 cofactor and inhibits the transcriptional activation of SIX1 and EYA1, which changes the developmental process of ear follicles and leads to craniofacial cartilage defects. Therefore, Sobp was determined as a candidate gene for BOR syndrome and other deafness syndromes [94]. Sox^2+^ proneurosensory progenitors within otic ectoderm form sensory cells and neurons for hearing. EYA1-SIX1 signaling facilitated the binding of Brg1 to enhancers of Sox2, which was correlated with the differentiation of neurosensory cells within the otic ectoderm [167]. Depleting Brg1 or EYA1-SIX1 signaling inhibited the expression of Sox2, and the subsequent deficiency of neurosensory identity resulted in the aberrant apoptosis of otic neurosensory cells [167]. The SIX1 mutant fetal mice display primary defects in myogenesis and muscle [95]. Pairing box 3 (Pax3) plays an important role in the development of lower axons and limb muscles [168]. The overexpression of SIX1 not only increased the expression of Pax3, but also elevated the proliferation of Pax7 ( +) cells by up-regulating Smad1/5/8 [169, 170]. EYA1 activates multiple target genes, including Pax3, MyoD, and myogenin. Consequently, EYA1/EYA2 mutant mice show delayed myogenesis [14, 171]. Lee et al. confirmed that the four transcription factors, SIX1, EYA1, Esrrb, and Pax3, transform fibroblasts into induced myogenic stem cells (iMSCs). Moreover, those iMSCs were effectively different into multinucleated myotubes and had a stronger proliferative capacity than muscle-derived stem cells. Thus, iMSCs provide a new therapy strategy for muscle atrophy and muscle regeneration [172].

### Lung

Asthma is a chronic disease caused by inflammation in the respiratory tract [173]. Yang et al. identified that SIX1 was up-regulated in a chronic asthma murine model. In addition, with SIX1 knockdown, the expression and secretion of MMP-2 and MMP-9, mediators related to airway remodeling, were reduced, and the activation of the NF-KB pathway in the lung was simultaneously suppressed, which effectively suppressed airway inflammation and remodeling in asthmatic mice [59]. In addition, studies have also found that miR-448-5p overexpression inhibited TGF-β1-induced EMT and pulmonary fibrosis in asthmatic mice by targeting SIX1 expression. The results suggested that the miR-448-5p/TGF-β1/SIX1 axis may be a novel target for strategies to prevent airway remodeling in asthma [174]. Idiopathic pulmonary fibrosis (IPF) is a chronic, progressive, and fibrotic lung disease. Increased expressions of SIX1 and EYA were observed in IPF lung tissue samples and IPF-derived alveolar type II cells (AT2). SIX1-driven expression of macrophage migration inhibitory factor (MIF) was attributed to the process of lung fibrosis. ISO-1, a MIF inhibitor, reduces SIX1-mediated cell proliferation and the expression levels of α-SMA and Col1a1, providing a potential pathway for IPF therapy [58].

### Hematologic cells

It is known that the GATA and PAX-SIX-EYA-DACH transcriptional networks (PSEDNs) are essential for proper development. Creed et al. indicated that SIX1 and SIX2 strengthened GATA1 function and accelerated erythropoiesis in human erythroleukemia cell lines and primary hematopoietic stem-progenitor cells (HPCs). Conversely, the erythropoietin (EPO)-stimulated erythropoiesis process was impaired when SIX1 was knocked out [60]. MLL encodes a histone methyltransferase that maintains key gene expressions during embryonic development and hematopoiesis. MLL-AF9 is known to be a driver of acute myeloid leukemia (AML). Zhang et al. confirmed that SIX1 was the target gene of leukemic initiating cells (LICs) confusion protein (MLL-AF9), and blocking WNT/SIX1 signaling by Wnt signaling inhibitors suppressed the progression of AML [61]. EYA1 and SIX1 are induced by MLL-ENL, EYA1 could immortalize hematopoietic progenitor cells, and SIX1 enhanced the transforming capacity of EYA1 [175].

### Others

Immune disorders are associated with non-classical NF-KB pathway dysfunction, and expression of NF-κB-inducing kinase (NIK) is the rate-limiting step in non-canonical NF-κB activation [176, 177]. NIK activates SIX protein in macrophages. SIX1 and SIX2 are identified as integral components of the non-canonical NF-κB signaling cascade. SIX1 and SIX2 inhibit the trans-activating function of RELA and RELB, the inflammatory gene promoters, leading to sustained non-classical NF-KB pathway activation. In addition, this research illustrated that SIX1 suppressed inflammation and promoted recovery ability from endotoxic shock in mice. Moreover, SIX1 and SIX2 preserved RAS/P53-driven non-small cell lung cancer from SMAC mimetic chemotherapeutic drug-induced inflammation [178]. Thus, NIK-SIX signaling axis programs inflammatory gene expressions in both physiological and pathological conditions.

### The potential therapeutic targets of RDGN

It is generally accepted that cancer drugs can target particular molecular proteins to inhibit the malignant behavior of cancer cells, such as BCL2 [179–182]. Currently, molecular target therapeutics are mainly inhibitors of oncogenes, and it's challenging to design or discover an effective cancer drug targeting tumor suppressor [183–185]. The changes in RDGN members' expression and physiological functions have been observed in various tumors. Moreover, a series of studies found that the aberrant expression of RDGN contributed to the malignant behaviors of tumors, as discussed in the previous sections. Several studies have proved that targeting RDGN members maybe a potential therapeutic approach for cancer therapy [86]. Here, we summarize potential therapeutic strategies targeting RDGN (Fig. 4).

### DACH1

Studies have extensively demonstrated that the down-expression of DACH1 is related to poor prognoses in many tumors, such as BC, prostate cancer, lung cancer, and hepatocellular carcinoma [8, 103, 105, 186–188]. Moreover, Nuclear DACH1 expression could be a luminal biomarker and independent prognosis factor [189]. Importantly, circulating cell-free DNA-based four methylation markers (RASGRF1, CPXM1, HOXA10, and DACH1) and two parameters (cfDNA concentration and the mean of 12 methylation markers) could detect early breast cancer. The sensitivity and specificity are comparable to mammography screening [190]. Therefore, DACH1 can be used as a potential molecule in cancer diagnosis and therapy.

Chu et al. also found that the reactivation of DACH1 might be a novel renal clear cell cancer therapy because it suppressed the clear renal cell cancer cell growth and proliferation by down-expressing the cyclin D1 [34]. The overexpression of miR-217 enhanced cell cycle progression to promote the proliferation of BC cells, and DACH1 is one of the targets of miR-217. Therefore, targeting miR-217/DACH1 axis is a plausible strategy for BC therapy [191]. Liu et al. found that DACH1 was negatively correlated with CXCL8 via evaluating the lung adenocarcinoma cell lines and tissues. Meanwhile, it had been demonstrated that AP-1 and NF-kB activation sites of CXCL8 promoter were repressed by DACH1. This result demonstrated that the DACH1-CXCL8 axis regulated lung cancer progression and affected the prognosis of lung adenocarcinoma patients [28]. However, there are studies showing that DACH1 plays an oncogenic role in endometrial cancer and leukemia [76, 104]. Similarly, Hu et al. showed that DACH1 could be identified as a tumor promoter based on the organoid model. Overexpression of DACH1 increased stem cell proliferation by suppressing bone morphogenetic protein (BMP) via SMAD4. Therefore, they proposed that DACH1 could be a potential therapeutic target for CRC patients [17]. This discrepancy may attribute to the activation of specific signaling pathways by DACH1 and the heterogeneity in different organs.

Restoration of DACH1 expression may be an innovative approach to cancer treatment. For example, the restoration of DACH1 expression could sensitize HCCs to 5-fluorouracil [36], and it also enhanced the sensitivity to docetaxel in CRC cells and gastric cancer cells [18, 33]. 5-Aza-2'-deoxyazacytidine, a demethylating agent, reactivated DACH1 expression, which would manipulate for tumor therapy [33]. Furthermore, a recent study indicated that DACH1 methylation might be an early marker in esophageal cancer and correlated with progression. DACH1 hyper-methylation was associated with poor differentiation and advanced stage. Ectopic expression of DACH1 activated TGF-β signaling and inhibited cancer cell proliferation and tumor growth. Therefore, restoration of DACH1 by demethylase may be a promising therapeutic target for patients with esophageal cancer [31]. A similar phenomenon is also observed in lung adenocarcinoma cells [192].

Metformin, a common medicine for diabetes, possesses an anti-tumor role in several kinds of cancer cells, such as BC and pancreatic cancer [193, 194]. The study showed that metformin increased the expression of DACH1 primarily via activating AMPK phosphorylation, then DACH1 inhibited the secretion of CXCL1 though the inhibition of NF-kB in esophageal squamous carcinoma cells. Thus, the antitumor effect of metformin was attributed to the reduced PMN-MDSC accumulation in the tumor microenvironment via AMPK-DACH1-CXCL1 signaling [32].

### SIX1

Members of the SIX family, especially SIX1, play oncogenic roles in various cancers, which suggests that SIX1 may be a potential therapeutic target in cancer therapy.

SIX1 promoted stem or progenitor cell phenotype and induced EMT in tumor cells, indicating that SIX1 knockdown may be a method of inhibiting tumor growth and delaying tumor progression [98]. The study showed that SIX1 strengthened the special subgroup of cancer stem/progenitor cells via binding to transcriptional factors [76, 98]. Several studies in recent years have demonstrated that targeting the SIX/EYA transcriptional complex delayed tumor progression, indicating that the inhibitors’ development of this complex could be effective in cancer therapy [96]. Meanwhile, Zhou et al. found a small molecule SIX1/EYA inhibitor compound named NCGC00378430, which effectively suppressed the metastasis of BC in the murine model [195]. It’s been widely demonstrated that SIX1 was over-expressed in prostate cancer. The study has found that the GRP75-USP1-SIX1 complex inhibited the growth of prostate cancer. Moreover, this compound could also alleviate the drug resistance effect of prostate cancer cells in mouse models, which may provide novel ideas for the future treatment of prostate cancer [196]. Many studies have illustrated that aerobic glycolysis accelerated tumor growth. SIX1 was found to boost the ability of aerobic glycolysis. Meanwhile, miRNA-548a-3p was shown to be negatively correlated with SIX1 and could inhibit the glycolytic function of SIX1. This research showed that targeting the miRNA-548a-3p/SIX1 axis would presumably serve as a therapeutic target in BC [197]. The short-form recepteur d'origine nantais (sf-RON) was found to enhance glucose metabolism in gastric cancer by activating the β-catenin/SIX1 signaling axis, which promotes tumor cell proliferation, which indicated that sf-RON might serve as a promising therapeutic target for gastric cancer [198]. SNS-032 was able to induce the degradation of SIX1 through the inactivation of the EGFR-AKT-USP1 axis, and SNS-032 in combination with sorafenib was found to inhibit tumor growth in a mouse model of HCC [199]. Zhang et al. revealed that SIX1 was one of the WNT-controlled target genes in MLL-AF9-transformed LICs. MLL-AF9 was found to induce both the expression of SIX1 and EYA1 [175, 200]. MLL-AF9 promotes WNT/-linked protein-dependent growth of LICs by binding TCF7L2 to the TCF/LEF, a regulating element of SIX1. Furthermore, Zhang et al. illustrated that SIX1 directly affected the expression activity of SIX1 and EYA1 in MLL-AF9. With the administration of WNT signaling inhibitors, WNT/SIX1 signaling was disrupted, which delayed the progression of AML [61]. Monteiro et al. proposed that the methylation of the SIX1 promoter could be an independent prognostic factor for overall survival in melanoma patients [201].

Liu et al. fund that SIX1 expressions were high in human tumor samples and inversely correlated with immune cell infiltration in the TME. Tumor growth was decreased in an immune-dependent way, and the anti-tumor immunity was strengthened in the tumor microenvironment with the silence of SIX1. These results suggested that targeting SIX1 provided a novel idea for cancer immunotherapy [202].

### EYA

EYA is a protein tyrosine phosphatase that facilitates the repair of DNA damage and tumorigenesis. Therefore, it is speculated that EYA may lead to relapse from chemotherapy and radiotherapy [86, 89]. And we propose that using EYA inhibitors may reduce chemo-resistance. Recently, it has been found that EYA2 Tyr phosphatase inhibitors could reduce the mesenchymal phenotype, enhance the immunogenicity of tumor cells, weaken tumor growth, and increase the antitumor effects of anti-PD-1 in the mouse BC model by targeting FBOX7. This experiment may provide a favorable basis for immune checkpoint block therapy via combination with FBOX7/EYA2 inhibitors [203]. Moreover, Vartuli et al. discovered that EYA3 was highly expressed in BC and reduced the number of infiltrating CD8^+^T cells along with T-cell depletion. Meanwhile, EYA3 was able to enhance PD-L1 expression via c-Myc to accelerate tumor growth. Hence, it is believed that the small molecule inhibitors of EYA3 could improve the curative effect of immune checkpoint blockades through their combination with an immune checkpoint inhibitor [138]. The overexpression of EGFR is associated with poor prognosis in BC. MiR-338-3p inhibited EGFR and subsequently activated EYA2, which accelerates BC tumor cell growth and lung metastasis. Moreover, the application of EYA2 inhibitors or miR-338-3p activators may be a promising therapeutic approach for BC lung metastasis [204]. The binding of EYA2 and DACH1 transcriptionally regulates the expression of SOCS3 and inhibits the progression of hepatocellular carcinoma by blocking the activation of the SOCS3-mediated JAK/STAT signaling pathway. Meanwhile, the delivery of EYA2 can be used to treat orthotopic liver cancer in nude mice, which showed that EYA2 may be a target for liver cancer treatment [205]. EYA4, a member of the EYA family, has been identified as a promising tumor suppressor in HCC, potentially inhibiting malignant behaviors in HCC cell lines by interfering with NF-κB/RAP1 signaling activation [145]. EYA4 was also identified as a tumor suppressor and abnormally hypermethylated in ESCC [206]. A class of reversible EYA inhibitors has been elucidated that selectively inhibit the activity of EYA2 phosphatase, and such compounds inhibit tumor cell migration, such as MLS00054460, MLS000585814, NCGC00249327, and NCGC00241224. And they could serve as prototypes for the development of EYA2 phosphate-specific anticancer drugs [207]. ​Moreover, another small-molecular complex was NCGC00249987, which prevented Mg^2+^ to bind with EYA2, and further inhibited the activity of EYA Tyr phosphatase. Anantharajan et al. indicated that NCGC00249987 specifically suppressed the migration and invasion in lung adenocarcinoma cells, but it didn't inhibit the survival and proliferation or of cancer cells [93].

### Perspective

The RDGN members determine the biological behavior and therapeutic response of tumor cells and also affect the development of organs and tissues. In this work, we review the structures and functions of the RDGN gene family in tumorigenesis. We also summarize their roles in the physiological processes of tissue specification and organ development. The RDGN family has a specific expression pattern in tumors, which is associated with the clinicopathologic features and prognosis of cancer patients. There are interactions among RDGN family and a variety of tumor-related signaling pathways. These complex regulatory networks suggest that the functions of the RDGN members could be regulated by existing small-molecule targeted therapies, such as EGFR and Wnt inhibitors. In addition, the RDGN family is involved in the regulation of the tumor microenvironment and immune cell functions, shedding new light on tumor immunotherapy. However, the mechanism by which the RDGN family regulates tumorigenesis and its regulatory relationship with other molecules remain unclear.

The RDGN family regulates the expansion and differentiation of organ-specific stem cells or progenitor cells. Thus balanced expressions of RDGN determine the normal development of multiple organs. Correspondingly, their dysfunctions attribute to a series of human diseases, such as CKD, coronary disease, and lung fibrosis. With the rapid advancement of gene editing techniques, targeting RDGN for gene therapy is plausible.

From organ development to tumorigenesis, the RDGN is a conserved network. DACH1 hyper-methylation results in epigenetic silencing in various cancers, including esophageal and breast cancers. Restoring DACH1 expression may inhibit tumorigenesis via demethylase. SIX1 is overexpressed in many cancers compared to normal tissue. Inhibition of SIX1 by small interfering RNA may suppress the progression of cancer. As a co-transcriptor for SIX1, the tyrosine phosphatase activity of EYA promotes the progression of tumors. It has been confirmed that this activity could be specifically inhibited by benzbromarone and benzarone. At present, the major studies on DACH1, SIX1, and EYA have been focused on their individual biological function and underlying molecular mechanisms. The multi-omics study is needed to explore the complex interaction of RDGN and organ-specific function in different cancers.

With the development of precision medicine and genomics, molecularly targeted therapies have revolutionized the treatment strategy for several kinds of cancers. At present, researches on RDGN are still in the preclinical stage. Translational potential needs to be explored to address the clinical value of targeting the RDGN network for cancer therapeutic strategy.

## Data Availability

Not applicable.

## Abbreviations

RDGN: Retinal determinant gene network
TF: Transcription factor
CKD: Chronic kidney disease
CHD: Coronary heart disease
EMT: Epithelial-mesenchymal transition
CSCs: Cancer stem cells
DD1: Dachshund domain 1
DD2: Dachshund domain 2
HD: Homeoprotein domain
SD: SIX domain
ER-α: Estrogen receptor-α
BOR syndrome: Branchio-oto-renal syndrome
KLF5: Kruppel-like factor 5
YB-1: Y-box binding protein 1
BLBC: Basal-like breast cancer
RSK2: Ribosomal S6 kinase 2
RPL26: Ribosomal protein L26
HCC: Hepatocellular carcinoma cell
CRC: Colorectal cancer
MMP-9: Matrix metalloproteinase 9
DKD: Diabetic kidney disease
ESRD: End-stage renal disease
ANNA3: Antineuronal nuclear antibody 3
CaMKII: Calcium/calmodulin-dependent protein kinase II
HDAC4: Histone deacetylase 4
tPA: Tissue-type plasminogen activator
Sobp: So-binding protein
Pax3: Pairing box 3
MIF: Migration inhibitory factor
EPO: Erythropoietin
AML: Acute myeloid leukemia
BMP: Bone morphogenetic protein
sf-RON: Short-form recepteur d'origine nantais
